# Amino Acid Transport Defects in Human Inherited Metabolic Disorders

**DOI:** 10.3390/ijms21010119

**Published:** 2019-12-23

**Authors:** Raquel Yahyaoui, Javier Pérez-Frías

**Affiliations:** 1 Laboratory of Metabolic Disorders and Newborn Screening Center of Eastern Andalusia, Málaga Regional University Hospital, 29011 Málaga, Spain; 2 Grupo Endocrinología y Nutrición, Diabetes y Obesidad, Instituto de Investigación Biomédica de Málaga-IBIMA, 29010 Málaga, Spain; 3 Grupo Multidisciplinar de Investigación Pediátrica, Instituto de Investigación Biomédica de Málaga-IBIMA, 29010 Málaga, Spain; jpf@uma.es; 4 Departamento de Farmacología y Pediatría, Facultad de Medicina, Universidad de Málaga, 29010 Málaga, Spain

**Keywords:** SLC, solute carriers, membrane transport, inborn errors of metabolism, amino acid transporter, symporter, inherited metabolic disorders

## Abstract

Amino acid transporters play very important roles in nutrient uptake, neurotransmitter recycling, protein synthesis, gene expression, cell redox balance, cell signaling, and regulation of cell volume. With regard to transporters that are closely connected to metabolism, amino acid transporter-associated diseases are linked to metabolic disorders, particularly when they involve different organs, cell types, or cell compartments. To date, 65 different human solute carrier (SLC) families and more than 400 transporter genes have been identified, including 11 that are known to include amino acid transporters. This review intends to summarize and update all the conditions in which a strong association has been found between an amino acid transporter and an inherited metabolic disorder. Many of these inherited disorders have been identified in recent years. In this work, the physiological functions of amino acid transporters will be described by the inherited diseases that arise from transporter impairment. The pathogenesis, clinical phenotype, laboratory findings, diagnosis, genetics, and treatment of these disorders are also briefly described. Appropriate clinical and diagnostic characterization of the underlying molecular defect may give patients the opportunity to avail themselves of appropriate therapeutic options in the future.

## 1. Introduction

Amino acids are key metabolites that are vital for cells and organisms. They are used as energy metabolites, are the basic elements for protein synthesis, and are precursors or aid in the biosynthesis of signaling molecules such as neurotransmitters and peptide hormones. Amino acids also play a role in the synthesis of many other molecules, such as nucleotides, polyamines, glucosamines, creatine, and related carbohydrates. In addition, amino acids are the main generators of C1 compounds (one-carbon molecules) [1]. Amino acid transporters play very important roles in nutrient uptake, neurotransmitter recycling, protein synthesis, gene expression, cell redox balance, cell signaling, and regulation of cell volume [2].

Amino acids have very diverse chemical natures and structures. In mammalian genomes, amino acid transporters are classified into solute carrier (SLC) families based on sequence similarity [3]. Members of sequence-related families may share an affinity for a substrate class and/or driving force. It appears that the combination of a certain amino acid and a coupling substrate is not exclusive, e.g., the same amino acid can be transported by different mechanisms. Environmental conditions have an effect on amino acid transport systems. These transport systems adapt to specific conditions by selecting the particular coupling mode that permits them to achieve the affinity necessary for certain physiological conditions [3,4].

To date, 65 different SLC families and more than 400 transporter genes have been identified. Eleven families are known to include amino acid transporters [1]. Despite the fact that most amino acid transporters have already been identified and characterized, there continues to be a substantial number of orphan transporters including in some SLC families. Amino acid transporters have recently been categorized into different systems depending on functional criteria, such as Na^+^-dependence and substrate preference, transport mechanism, and regulatory properties [5,6,7,8,9,10,11,12,13,14].

In general, intracellular amino acid concentrations are greater than—or at least equal to—the concentrations found in extracellular fluid. In order to concentrate amino acids within the cell, active transport mechanisms are used. Multiple amino acid transporters (e.g., systems A, N, and XAG) combine thermodynamically competent Na^+^ influx with amino acid flux to catalyze amino acid uphill transport via secondary active transport [5,6,7,8]. Na^+^/K^+^-ATPase activity contributes to maintaining the Na^+^ transmembrane gradient well out of equilibrium. Furthermore, some transport activities are also linked to gradients of other ions, including K^+^, H^+^, and OH^−^ [6]. In the system y+, the electrical component of the transmembrane potential difference is the driving force for cationic amino acid influx. Several different transport systems may cause uptake of a specific amino acid into a particular cell type [9,11,15,16]. For example, at least two secondary active transporters, known as system A and system N, as well as the system ASC (Ala/Ser/Cys) exchanger can lead to glutamine being transported into hepatocytes [11]. Some members of the SLC6 family that transport neurotransmitters are Na^+^/Cl^−^ dependent. Other systems related to amino acid transport are systems B^0^ (transport for most neutral, branched, and aromatic amino acids), system L (Na^+^-independent transport system for neural amino acids), and system T (Na^+^-dependent taurine uptake).

An association has been found between amino acid transporter-associated diseases and metabolic disorders in instances in which transporters are closely connected to metabolism, particularly when they involve different cell types, organs, or cell compartments [17,18,19]. Table 1 summarizes the human SLC amino acid transporters that have been identified and their respective transporter genes. In this work, the physiological functions of amino acid transporters will be described by the inherited diseases that arise from transporter impairment. The name of the amino acid transporter, gene/locus, location, phenotype MIM number, inheritance, and main clinical manifestations of these inherited metabolic diseases are summarized in Table 2.

This review intends to summarize and update all the conditions in which a strong association has been found between an amino acid transporter and an inherited metabolic disorder. Many of these inherited disorders have been identified in recent years. The pathogenesis, clinical phenotype, laboratory findings, diagnosis, genetics, and treatment of these disorders are also briefly described.

## 2. Dicarboxylic Aminoaciduria (OMIM #222730)

Dicarboxylic aminoaciduria (DA) involves excess excretion of acidic amino acids (aspartate and glutamate) in the urine due to incomplete anionic amino acid reabsorption from the glomerular filtrate in the kidney [20]. It is a rare autosomal recessive disorder caused by loss-of-function mutations in the *SLC1A1* gene, which encodes the excitatory amino acid transporter 3 (EAAT3). This affects glutamate and aspartate transport in the intestine, kidney, and brain [21]. It was first described in 1974 [22]. EAAT3 is widely expressed in the brain’s cortex, especially in the hippocampus, basal ganglia, cerebellum, and olfactory bulb. It is believed to be involved in the biosynthesis of glutathione and GABA [20,23]. Dicarboxylic aminoaciduria has an estimated incidence of 1:36,000, according to the results of the newborn urine-screening program that has been carried out in Québec for more than 30 years [24,25].

This disorder’s clinical features have not been widely studied. However, an association with mental retardation has been reported in two cases and with kidney stones in one case [20,26]. It was believed to be a mostly asymptomatic condition [27], but more recent studies have shown that DA may be linked to the pathogenesis of obsessive-compulsive disorder (OCD) and schizophrenia spectrum disorders [28,29,30,31,32,33,34].

## 3. Epileptic Encephalopathy SLC1A2-Related (OMIM #617105)

It has recently been reported that heterozygous de novo mutations in *SCL1A2* underlie severe early-onset epileptic encephalopathy [35]. *SLC1A2* encodes excitatory amino acid transporter 2 (EAAT2), which is one of the major glutamate transporters that are expressed in astroglia. The role of SLC1A2 is to clear glutamate from the extracellular space at the synapse [36]. Excessive glutamate levels can result in neurotoxicity, which may be a cause of epilepsy [37]. The few cases that have been described involve an extremely severe phenotype that is characterized by the onset of seizures in the first week after birth and profound impairment of development with no detectable regression. All cases presented with multiple seizure types, including prominent tonic and myoclonic seizures, as well as spasms. One case is presented with profound growth failure and microcephaly [35,37,38].

A recent study has demonstrated that the three known variants involved in this condition are localized in the trimerization domain of SLC1A2. The Leu85Pro variant reduces wild-type SLC1A2 protein localization and function via a dominant negative mechanism [39]. A recent study suggests that this disorder can be caused not only by de novo mutations, but also by biallelic variants in *SLC1A2*. Furthermore, there is also a recessive mode of inheritance [37]. Although the β-lactam antibiotic ceftriaxone has been demonstrated to increase expression of EAAT2 [40] by increasing EAAT2 promoter activation [41], the efficacy of ceftriaxone continues to be controversial [37,42].

## 4. Episodic Ataxia Type 6 SLC1A3-Related (OMIM #612656)

Episodic ataxia type 6 (EA6) is caused by mutations in *SLC1A3*. This gene encodes excitatory amino acid transporter 1 (EAA1), which is a glial glutamate transporter that clears glutamate from the synaptic cleft and regulates neurotransmitter concentrations at excitatory glutamatergic synapses [43]. Heterozygous mutations in *SLC1A3* are associated with a broad range of clinical phenotypes. To date, a total of seven patients from four families have been reported to have infantile episodic ataxia and seizures, migraine, dysarthria, intellectual disability, intentional tremor, and, in some cases, alternating hemiplegia [44,45,46,47]. Onset typically occurs in early childhood. It is common for episodes to be triggered by emotional and physical stress [48]. The clinical phenotypes differ from each other according to glutamate reuptake ability [44,45]. Studies of in vitro functional expression in COS-7 cells have found that mutant EAAT1 leads to a reduction in glutamate uptake compared to the wild-type channel [45]. It has recently been reported that two affected members of the same family have been showing adult-onset ataxia with dysarthria and jerky ocular pursuit movements. They had a good response to acetazolamide [43]. Acetazolamide is a carbonic anhydrase inhibitor that is particularly effective in episodic ataxia type 2 caused by *CACNA1A* gene mutations [48].

## 5. Spastic Tetraplegia, Thin Corpus Callosum, and Progressive Microcephaly Linked to SLC1A4 (OMIM #616657)

*SLC1A4* is a neuronal and glial gene that encodes the alanine/serine/cysteine/threonine and glutamate transporter 1 (ASCT1) [49]. ASCT1 transports L-serine across the blood-brain barrier from astrocytes to the extracellular space, and, lastly, into neurons [50,51]. Biallelic mutations in the *SLC1A4* gene have been implicated as a very rare cause of neurodevelopmental disorders, including congenital microcephaly, epileptic encephalopathy, severe hypotonia, global developmental delay, spasticity that is most prominent in the lower limbs, and thin corpus callosum [49,52,53,54,55]. At present, this genetic condition has been diagnosed almost exclusively in a small number of individuals of Ashkenazi Jewish descent. There is a high prevalence of heterozygous healthy carriers in this population [52].

## 6. Cystinuria (OMIM #220100)

Cystinuria is a common genetic disorder caused by defective transport of cystine and dibasic amino acids (ornithine, lysine, and arginine) across the apical membrane of epithelial cells in the renal proximal tubules and the gastrointestinal tract mediated by the b^0,+^ transporter [21,56,57,58,59,60,61,62,63,64,65,66]. The b^0,+^ transporter is a heterodimer comprising rBAT (encoded by *SLC3A1*) and b^0,+^ AT (encoded by *SLC7A9*) subunits linked by a disulfide bridge [67,68,69,70]. Hyperexcretion of cystine and dibasic amino acids in the urine is this disease’s biochemical hallmark [21]. Global prevalence of cystinuria is estimated to be 1:7000, which ranges from 1:2500 in Libyan Jews (due to the founder effect) to 1:100,000 in Sweden [59,61,71].

Cystine is the least soluble of the four amino acids. As such, it can crystallize in the urinary tract, which results in the formation of cystine stones in the kidney and, less frequently, in the bladder [71,72]. It has been estimated that 1–2% and 6–8% of urinary tract stones in adults and children, respectively, are caused by cystinuria and, thus, in the pediatric population, cystinuria is the most common genetic cause of urolithiasis [73,74,75]. Though stones may form at any age, it most commonly occurs in the first two decades of life. Furthermore, 75% of patients develop bilateral stones [71,76,77]. In general, men are affected more severely than women and have a higher number of stones [61]. A diagnosis is usually established by confirming that stones are composed of cystine [66]. Cystine stone formation may lead to complications such as obstructive uropathy and pyelonephritis. Up to 70% of patients may develop chronic kidney disease [76,78,79].

Cystinuria is believed to be an autosomal recessive disorder, even if no mutations have been identified or only one mutant allele has been found, which is the case in approximately 5% of patients with cystinuria [80,81]. Based on genotyping, cystinuria can be classified as Type A (caused by mutations in *SLC3A1*) or Type B (caused by mutations in *SLC7A9*) [82]. In cystinuria type A, heterozygous individuals have a normal amino acid urinary pattern. In cystinuria type B, heterozygous individuals usually have increased cystine and dibasic amino acid urinary excretion and may develop nephrolithiasis. Thus, it can be considered an autosomal dominant trait [83]. Manifesting heterozygous individuals suggest that cystinuria type B can be transmitted in an autosomal dominant inheritance pattern with incomplete penetrance [84]. Global prevalence and disease severity appear to be the same in cystinuria types A and B [66]. It is possible that there is a very rare third type of cystinuria caused by a mutation in each of the two previously mentioned genes (cystinuria type AB), but it still needs to be confirmed in order to demonstrate the digenic inheritance of cystinuria [80]. Molecular analyses in some families have found the co-occurrence of a single gene with two mutations and another gene with one mutation, which is compatible with an AAB or BBA genotype [85,86]. To date, 247 mutations have been reported in *SLC3A1* and 160 mutations have been reported in *SLC7A9* in the Human Gene Mutation Database (HBMD^®^ Professional 2019, http://www.hgmd.cf.ac.uk/ac/index.php, accessed November 15, 2019) [87]. Newborn urine-screening programs may detect this disorder. Some of the cystinuria patients detected via Québec’s newborn screening program developed cystine stones in their first decade of life [88,89].

In order to treat cystinuria, cystine solubility is increased by boosting urine volume and pH. Though it can be difficult to achieve in young children, hyperdiuresis is the most important component in the prevention of cystine stone formation [60,61,90]. For the most severely affected patients, D-penicillamine and tiopronin thiols are treatment options [60,91]. These sulfhydryl drugs convert cystine into cysteine-penicillamine or cysteine-tiopronin, respectively, which are more soluble mixed disulfides. However, these drugs are associated with significant side effects [92,93].

The association of type A cystinuria with hypotonia and further clinical symptoms (hypotonia-cystinuria syndrome, OMIM #606407) is caused by a homozygous deletion on chromosome 2p21, which disrupts the *SLC3A1* gene [94,95,96,97,98,99]. Recently, aspartate/glutamate transporter 1 (AGT1), encoded by *SLC7A13*, has been identified as the second partner of rBAT. AGT1 is localized on the apical membrane of the S3 segment of the proximal tubules, where it forms a heterodimer with rBAT [100]. It has yet to be determined if *SLC7A13* mutations could help explain the isolated cystinuria observed in patients who still do not have any detectable mutation in *SLC3A1* and *SLC7A9* [101].

## 7. Hyperekplexia 3 (OMIM #614618)

Hyperekplexia is a potentially treatable and rare neurological disorder that is characterized by an exaggerated startle reflex caused by external stimuli (noise or touch) and generalized muscle stiffness. It presents in neonates immediately or soon after birth [102,103]. Hyperekplexia has been discovered in genetic screening studies to be genetically heterogeneous. The primary cause has been determined to be several missense and nonsense mutations in the postsynaptic glycine receptor (GlyR) alpha-1 subunit gene (*GLRA1*). More recently, a presynaptic component to this disease has been demonstrating with missense, nonsense, and frameshift mutations identified in the glycine transporter GlyT2 gene, *SLC6A5* (hyperekplexia 3) [104]. GlyT2 is mainly expressed in glycinergic neurons in the spinal cord, where it is present in the pre-synaptic membrane, which recaptures released neurotransmitters. In order to extend the amount of time glycine remains in the synaptic cleft and, thereby, activate glycine receptors more strongly, this transporter can be blocked. This would, in turn, enhance inhibition, and explain the stiffness observed after being startled [21,105]. The transmission pattern is consistent with autosomal recessive inheritance, even though some patients present with an autosomal dominant inheritance pattern [102].

Although the symptoms of hyperekplexia have been clearly defined, it may be mistaken for neonatal epilepsy and, consequently, diagnosis may be delayed. One aspect that does distinguish this disease from epileptic seizures is the fact that patients with hyperekplexia remain conscious during the tonic-jittery attacks [103]. Symptoms usually decrease during the first years of life, but the excessive startle response can continue well into adulthood. This can lead to unprotected falls, which may cause serious injuries [106]. Hyperekplexia can cause serious consequences in some patients, including sudden infant death due to episodes of apnea and/or brain damage [21]. It is much less likely that patients with hyperekplexia who have a benign phenotype and *SLC6A5* variants will develop recurrent infantile apnea compared to patients who have *GLRA1* variants. On the other hand, developmental delays are more alike in patients with *SLC6A5* variants than in those with *GLRA1* variants [107]. Hyperekplexia management may include medication with an allosteric potentiator of the inhibitory GABA_A_ receptor, clonazepam [103]. It has also been demonstrated that stimulation of P2X purinergic receptors with βγ-methylene adenosine 5′-triphosphate induces upregulation of GlyT2 transport activity via an increase in total and plasma membrane expression as well as a reduction in transporter ubiquitination [108]. Another therapeutic strategy is maternal soothing, which has been observed to halt hyperekplexia attacks on numerous occasions [103].

## 8. Retinal Dystrophy SLC6A6-Related

The retina is one of the tissues in the body with the greatest amount of taurine because of high concentrations in the photoreceptor layer. Taurine concentration peaks during retina formation and remains high in adulthood, which indicates a role in both photoreceptor development and maintenance [109]. The transmembrane sodium-dependent taurine transporter (TAUT) transports nutritional taurine into retinal pigment epithelium cells, which supply it to the photoreceptor cells [110]. A recent study on a consanguineous family reported that a biallelic mutation of human *SLC6A6*, which encodes the TAUT, is linked to early-onset pan-retinal degeneration. Functional studies have demonstrated that ^3^H-taurine uptake by peripheral blood mononuclear cells was reduced by 95% and taurine levels were severely reduced in plasma, skeletal muscle, and the brain. Extraocular dysfunctions that were present in *Slc6a6*^−/−^ mice have not been observed in the studied patients, but significantly increased urinary excretion of 8-oxo-7,8-dihydroguanosine indicated to be generally enhanced oxidative stress and RNA oxidation [109]. Taurine substitution will likely not be effective because of taurine impaired renal reabsorption, but the use of taurine conjugates taken up into cells could be a promising therapeutic approach [109].

## 9. Glycine Encephalopathy with Normal Serum Glycine (OMIM #617301)

In 2016, a new type of nonketotic hyperglycinemia that arises from biallelic mutations in the *SLC6A9* gene, which encodes glycine transporter 1 (GLYT1), was reported [111]. GLYT1 is mainly located in the caudal regions of the brain (spinal cord, brain stem, and cerebellum), mainly in astrocytes [112]. It is key for the transport of glycine from the extracellular space and glycinergic transmission termination [113,114].

To date, only six cases from three families have been described in the literature [111,115]. The first case described was in a patient from Saudi Arabia and the other five were from Israel. All patients reported in the literature had neonatal disease onset, respiratory failure with a requirement for mechanical ventilation, severe hypotonia at birth and subsequent limb hypertonicity, and startle-like responses caused by tactile stimulation and sudden loud sounds. In addition, dysmorphic features, musculoskeletal system abnormalities (joint laxity, hip dislocation, hyperextension of knees, or other musculoskeletal abnormalities), abnormal antenatal findings, abnormal brain magnetic resonance imaging (MRI), high glycine concentrations in cerebrospinal fluid, and a high cerebrospinal fluid to plasma glycine ratio were also found in these patients [111,112,115]. The abnormal antenatal findings were cervical cysts, nuchal translucency, clenched fists, fourth ventriculomegaly, bilateral clubfeet, overriding toes, hyperextension of the knees, joint contractures, arthrogryposis, and hydrops fetalis [111,112,115]. In addition to the previously mentioned clinical findings, a diagnosis can be made based on high cerebrospinal fluid glycine levels, normal plasma glycine levels, an elevated cerebrospinal fluid to plasma glycine ratio, and genetic testing for *SLC6A9* mutations [112]. There is currently no curative treatment available. Disease management is exclusively supportive.

## 10. Mental Retardation, Autosomal Recessive 48 (OMIM #616269)

In 2015, a report was published on two families of different ethnic backgrounds with autosomal recessive intellectual disability who were studied via a combination of homozygosity mapping and exome sequencing [116]. Using these approaches, the authors identified two missense variants in the *SLC6A17* gene that segregated with the phenotype. The in silico and in vitro analysis provided evidence of the causal effects of the identified mutations [116]. The *SLC6A17* gene encodes a vesicular transporter protein known as XT1 or NTT4 [117]. This protein, which functions as a selective transporter of four amino acids (Pro, Gly, Leu, Ala) as well as Glu and is involved in regulating glutamatergic synapses, is mainly localized in the nervous system in glutamatergic and some GABAergic neurons [118,119,120,121].

Based on the two families’ clinical features, the phenotypic spectrum comprises moderate-to-severe intellectual disability with nearly no speech development, progressive tremors, and behavioral problems. Severely impaired motor development that may possibly prevent independent walking has also been observed. No distinctive facial phenotypes have been noted [116].

## 11. Hartnup Disorder (OMIM #234500)

The Hartnup disorder is an autosomal recessive inherited disorder caused by mutations in *SLC6A19*, which encodes the neutral amino acid transporter B^0^AT1 [122,123]. It affects neutral amino acid transport across the apical brush border membrane of the renal proximal tubule and intestinal epithelium, which leads to impaired intestinal uptake and tubular reabsorption of all neutral amino acids [124,125]. The transporter is associated with partner proteins, which are necessary for its expression. These partner proteins are collectrin in the kidney and angiotensin-converting enzyme 2 in the intestine, which are both components of the renin angiotensin system [126]. The major clinical symptoms of Hartnup disorder are pellagra-like dermatitis, intermittent cerebellar ataxia, and other neurologic and neuropsychiatric symptoms, which closely resemble those of dietary niacin deficiency [127,128]. A diagnosis can be made by confirming the characteristic excess of neutral amino acids in the urine and low-normal or normal plasma concentrations. Systemic tryptophan deficiency is key in the development of neuropsychiatric symptoms, given that tryptophan is the precursor of the neurotransmitter serotonin. Most importantly, a tryptophan deficiency leads to decreased niacin and nicotinamide availability, which may explain the pellagra-like dermatitis. This dermatitis responds to niacin supplementation [21,125]. The relationship between the physiopathological mechanisms of the disease and the ataxia present in some patients is still unclear [21].

Since this disease was first described in multiple members of the Hartnup family in 1956 [128], which is a considerable number of subjects who meet the biochemical diagnostic criteria have been reported. It is most often detected in newborn urine-screening programs [25]. Nevertheless, most patients are asymptomatic. Symptomatic patients usually develop skin lesions and neurological problems in early childhood. These symptoms tend to improve as the patient grows older [127]. Symptoms may be precipitated by sun exposure, diarrhea, fever, inadequate diet, or psychological stress. Patients occasionally present with mental retardation, seizures, or psychosis-like symptoms [129]. In order to prevent these symptoms, sufficient dietary intake of niacin and an adequate amount of tryptophan is necessary. Tryptophan ethyl ester has been used to successfully circumvent the transport defect [83]. Recently, a novel clinical and biochemical phenotype that mimics Hartnup disorder due to an X-linked *CLTRN* mutation (collectrin deficiency) has been reported [130].

## 12. Iminoglycinuria (OMIM #242600) and Hyperglycinuria (OMIM #138500)

The imino acids hydroxyproline and proline use the same renal tubular reabsorption mechanism as glycine. Iminoglycinuria is an autosomal recessive disorder that is expressed in homozygous individuals or individuals with combinations of mutated alleles that is associated with excessive urinary levels of proline, hydroxyproline, and glycine [21,131]. Several autosomal alleles including some that are partially expressed in heterozygous individuals cause the disorder. The molecular defect involves three genes: *SLC36A2*, which encodes the proton-dependent amino acid transporter PAT2, *SLC6A20*, which encodes the sodium-dependent imino acid transporter SIT1, and *SLC6A19*, which encodes the transporter B^0^AT1 [131]. *SLC36A2* appears to be the principal gene implicated in homozygous cases of iminoglycinuria [21]. Monoallelic or biallelic variants of the putative glycine transporter gene *SLC6A18* are also carried by some individuals (XT2), which denotes that these variants may also contribute to the phenotype [131].

A variety of clinical symptoms have been described in cases of iminoglycinuria, including hypertension, glycosuria, nephrolithiasis, mental retardation, atypical gyrate atrophy, deafness, and blindness. Given the lack of controlled prospective studies, these associations may be the result of ascertainment bias [131]. Large urine-screening studies have found iminoglycinuria to be a benign condition, with aminoaciduria only being detected retrospectively [21,25,124]. In newborns and infants less than six months old, iminoglycinuria is a normal finding [132].

Iminoglycinuria is generally considered to be the recessive phenotype whereas hyperglycinuria seems to be present in many but not all heterozygous individuals, which possibly presents as a dominant trait [131]. An investigation of intestinal transport indicates further complexity. Intestinal proline (but not glycine) transport is affected in some families whereas, in others, intestinal transport is unaffected [132].

## 13. Argininemia SLC7A2-Related

In 2019, the case of a Spanish newborn with high blood arginine levels found during newborn screening and two loss-of-function mutations in the *SLC7A2* gene was reported [133]. This gene encodes human cationic amino acid transporter 2 (CAT-2) [134]. Plasma and urinary cationic amino acids (Arg, Lys, and Orn) were high. Plasma and urinary ADMA levels as well as urinary guanidinoacetic acid levels were high at the time of diagnosis, but were normal at the two-year follow-up after following a protein-restricted diet. Currently, the child is five years old and remains asymptomatic, which presents with normal growth and psychomotor development. The disorder’s long-term natural history is not known, even though a low-protein diet is necessary to control amino acid and guanidinoacetic acid levels [133]. This disorder may potentially be detected in newborn screening while screening for arginase 1 deficiency (OMIM #207800).

## 14. Lysinuric Protein Intolerance (OMIM #222700)

Lysinuric protein intolerance (LPI) is a rare primary inherited aminoaciduria caused by biallelic mutations in the *SLC7A7* gene, which encodes the HAT light chain y^+^LAT1 [135]. In this disorder, there is defective transport of the dibasic cationic amino acids lysine, arginine, and ornithine at the basolateral membrane of epithelial cells in the renal tubules and small intestine, where the combination of y^+^LAT1 and 4F2hc generates an active amino acid transporter [135]. Impairment of intestinal absorption and renal reabsorption of dibasic amino acids causes a metabolic derangement that is characterized by low dibasic amino acid plasma concentrations and increased urinary excretion as well as urea cycle dysfunction, which leads to hyperammonemia and orotic aciduria [21].

The natural history of LPI has yet to be fully characterized and the clinical symptoms and severity of LPI vary. Breast-fed newborns and infants are usually asymptomatic. When formulas that have a higher protein content or supplementary foods that are high in protein are introduced, postprandial episodes of hyperammonemia usually emerge [136]. At around one year of age, patients commonly develop a strong aversion to high-protein foods with failure to thrive. In addition, the spleen and liver may be moderately enlarged [137]. Recurrent fractures occur frequently and rates of high, severe osteoporosis are observed from childhood to adulthood [138,139,140]. After excessive protein intake, patients with hyperammonemia may refuse to eat or vomit and present with stupor and drowsiness, which leads to a coma. These symptoms mimic those of urea cycle deficiencies. In many cases, hyperammonemia may be triggered by starvation, infection, and stress. Some patients only show transient mild hyperammonemia after meals [137,141]. Some patients may present with mild normochromic or hypochromic anemia, leukopenia, and thrombocytopenia. Aggravated viral infections, such as severe varicella infection, are frequent in LPI [142]. The poor growth and skeletal and immunological symptoms of LPI may be partially explained by the reduced availability of lysine, which is an essential amino acid. Renal disease is common in LPI. Disturbed proximal tubular function with mild proteinuria, tubular acidosis, and microhematuria may begin in childhood and often progress to glomerular dysfunction and end-stage renal failure [143,144,145]. Frequent complications include bone marrow involvement with hemophagocytic lymphohistiocytosis and interstitial pulmonary disease with alveolar proteinosis [137,146]. A few children and adults have died following a very uniform course of progressive multiple organ failure with interstitial lung involvement, progressive glomerulonephritis, and severe bleeding diathesis [147].

Diagnosing LPI is based on increased urinary excretion, low cationic amino acid plasma concentrations, especially lysine, and the identification of biallelic pathogenic variants in *SLC7A7* [148,149]. Finland has the largest number of patients who share the same founder mutation, c.895-2A > T, which causes a frameshift mutation and leads to a premature stop codon [150]. The main aims of LPI treatment are to prevent hyperammonemia and achieve normal growth and metabolism by providing a sufficient supply of protein and essential amino acids.

## 15. Retinitis Pigmentosa 68 (OMIM #615725)

The SLC7A14 transporter is mainly expressed in neural tissue and localized in lysosomes. It has been predicted that it mediates lysosomal uptake of cationic amino acids. *SLC7A14* mutations have been shown to cause autosomal recessive retinitis pigmentosa in Chinese patients [151]. SLC7A14-deficient mice have been observed to have an abnormal photoreceptor layer in the retina. In this layer, SLC7A14 expression increases as the retina develops and remains high in the mature retina [151]. Compound heterozygous missense mutations in the *SLC7A14* gene in an autosomal recessive retinitis pigmentosa case and a homozygous mutation in a case of Leber congenital amaurosis have been found in a recent study [152].

## 16. Deafness, Autosomal Dominant 25 (OMIM #605583)

In 2001, a large Czech family with nonsyndromic, slowly progressive high-frequency sensorineural hearing loss with postlingual onset and mutations in *SLC17A8* was reported [153]. The disorder segregated, which showed an autosomal dominant inheritance pattern. A second affected family, which was from the United States of America and of German descent, was described in 2008 [154] and a Korean family with the same phenotype was described in 2017 [155]. The *SLC17A8* gene encodes the vesicular glutamate transporter-3 (VGLUT3). This disorder may be produced by a specific defect in vesicular glutamate uptake and release, which is a process that is key in auditory coding at the sensory inner hair cell synapse [154,156]. An appropriate etiologic diagnosis of the genetic cause may give patients opportunities for appropriate therapeutic options in the future. In 2012, a study reported that delivering exogenous DNA by adeno-associated virus-mediated gene therapy restored hearing function in VGLUT3 knockout mice [157].

## 17. Epileptic Encephalopathy, Early Infantile 39 (OMIM #612949)

The aspartate–glutamate carrier 1 (AGC1) protein transports intramitochondrial aspartate to the cytoplasm in exchange for glutamate and a proton [158]. AGC1, which is encoded by the *SLC25A12* gene, is mainly expressed in the heart, skeletal muscle, and central nervous system [158]. It plays a role in the transfer of nicotinamide adenine dinucleotide (NADH)-reducing equivalents from the cytosol to the mitochondria. This transfer is crucial in connecting the glycolytic and oxidative phases of glucose catabolism [158]. Aspartate, which is produced in the mitochondria and exported to the cytosol, is critical for cell growth, proliferation, and synaptic transmission. Neuronal aspartate is continuously acetylated to form N-acetylaspartate (NAA) in the brain [159]. NAA is transported to glial cells, especially oligodendrocytes, where it is likely used to synthesize lipids and myelin [159].

Currently, there are a total of five patients with pathogenic *SLC25A12* variants described in the literature. They presented with a clinical phenotype consisting of arrested progressive encephalopathy, profound developmental delay, hypotonia, microcephaly, and myoclonic epilepsy [160,161,162,163]. In terms of neural abnormalities, two siblings described in the literature had cerebral atrophy, fluctuating basal ganglia changes, abnormal myelination, and decreased N-acetylaspartate levels, as indicated by magnetic resonance spectroscopy [160]. In another patient, global cerebral hypomyelination was noted on an MRI with an absence of gray matter, basal ganglia, brainstem, or spectroscopy-measured abnormalities [161]. One patient improved psychomotor functioning and resumed myelination by following a ketogenic diet [162]. In the oldest patient, who had MRI results from multiples ages, the clinical presentation was consistent with leukodystrophy of the leuko-axonopathy category instead of with a primary hypo-myelinating disorder [163].

## 18. AGC2 Deficiency (OMIM #605814, #603471)

Citrin deficiency is a rare autosomal recessive disorder that arises from a lack of function of the hepatic mitochondrial aspartate/glutamate transporter 2 (AGC2), which supplies aspartate to the cytosol for the argininosuccinate synthetase reaction, in which aspartate and citrulline condense to produce argininosuccinate [21,164,165]. Reduced cytosolic aspartate availability results in citrullinemia and hyperammonemia. This deficiency is caused by a homozygous or compound heterozygous mutation in the *SLC25A13* gene [165]. There are two principal clinical presentations, which are both age-dependent. Neonatal intrahepatic cholestasis caused by citrin deficiency (NICCD, OMIM #605814) and citrullinemia type II (CTLN2, OMIM #603471), which is present in adolescents and adults [166]. A third, less common presentation that may occur in childhood is failure to thrive and dyslipidemia caused by citrin deficiency (FTTDCD) [167].

Neonatal intrahepatic cholestasis with persistent jaundice, failure to thrive, hepatomegaly, and cholestasis has been observed in a large proportion of neonates with citrin deficiency. Anemia, low plasma albumin, and impaired coagulation with prolonged prothrombin time are frequent findings. Plasma citrulline is moderately increased and levels of other amino acids such as methionine, threonine, tyrosine, serine, or phenylalanine may be elevated [168,169,170,171]. These biochemical abnormalities may be detected via newborn screening [172].

CLTN2 can follow a period of normal health that lasts several decades. It is characterized by sudden onset of neuropsychiatric symptoms, recurrent ammonia intoxication that may lead to coma and death, and high levels of citrulline in plasma. Steatohepatitis and liver fibrosis are common complications [173]. Hyperammonemia develops in CTLN2 but not in NICCD due to the fact that liver ASS is significantly decreased in CTLN2 yet is normal in NICCD [164].

Hyperammonemic episodes in CTLN2 can be treated with IV sodium benzoate and sodium phenylacetate. Arginine should be administered and alcohol must be prohibited. Use of lactose-free or medium-chain triglyceride-enriched formula, a high protein/fat and low carbohydrate diet, and some medications (vitamin K_2_, lipid-soluble vitamins, and ursodeoxycholic acid) are used to treat NICCD. Sodium pyruvate may improve growth. The outcome of NICCD is generally good, with little intervention required by 12 months of age [174].

## 19. Hyperornithinemia-Hyperammonemia-Homocitrullinuria (HHH) Syndrome (OMIM #238970)

The HHH syndrome is caused by biallelic mutations in *SLC25A15*, which encodes the mitochondrial ornithine-citrulline carrier 1 (ORC1) [175]. The disease has a panethnic distribution. It is reported to be more prevalent in Canada, as a result of a founder mutation in Québec [176] in Italy [177] and in Japan [178]. This metabolic compartmentation disorder involves impaired importation of ornithine into mitochondria, which results in functional deficiency of both ornithine carbamoyltransferase (OTC) and ornithine aminotransferase (OAT). The intramitochondrial deficiency of ornithine leads to utilization of carbamoyl phosphate by pathways other than the one catalyzed by OTC, including homocitrulline formation from lysine and orotic acid formation secondary to excess flux down the pyrimidine biosynthetic pathway [179].

There is a broad spectrum of clinical manifestations that can present at any age, even though it usually manifests in early childhood. The most common symptoms are related to episodic hyperammonemia (confusion, lethargy, and coma) and neurological features such as developmental delay/intellectual disability, learning difficulties, signs of pyramidal tract dysfunction with spastic gait, cerebellar symptoms such as ataxia, and myoclonic seizures. Patients often present with liver dysfunction, which leads to reversible hepatocellular necrosis or acute hepatitis-like attacks and coagulopathy. In particular, there are reports of factor VII and X deficiencies [21,179,180]. The HHH syndrome may also have a more chronic, slowly progressive course with an aversion to high-protein foods, progressive encephalopathy with mental regression, and symptoms of motor dysfunction [179].

The differential diagnosis between the HHH syndrome and other hyperammonemic syndromes can be determined by the presence of the pathognomonic triad of hyperornithinemia, hyperammonemia, and homocitrullinuria. Treatment consists of preventing ammonia toxicity and a low-protein diet in addition to citrulline or arginine supplementation. Prognosis is variable. Some patients develop pyramidal dysfunction and spastic paraplegia [179,181].

## 20. Epileptic Encephalopathy, Early Infantile, 3 (OMIM #609304)

Biallelic mutations in the *SLC25A22* gene can cause early infantile epileptic encephalopathy-3 (EIEE3) [182,183]. *SLC25A22* encodes a glutamate/H^+^ symporter (GC1) that is highly expressed in the brain, especially in regions involved in motor coordination (red nuclei, olivary complexes, pontocerebellar fibers, and substantia nigra) [184]. GC1 is localized in the inner mitochondrial membrane and is more abundant in astrocytes, where it controls the uptake of glutamate. It catalyzes the glutamate/H+ symporter in order to transport glutamate into the mitochondria [184]. The dysregulation of extracellular glutamate and the activation of the extrasynaptic glutamate receptors would result from an absence of a functional SLC25A22 protein [185]. To date, 16 patients with EIEE3 have been reported [182,183,184,186,187]. Onset occurs in the first months of life and patients present with myoclonic seizures, severe global hypotonia, microcephaly, a “burst suppression” pattern in the interictal electroencephalogram, and an abnormal electroretinogram [184]. The prognosis of this condition is poor. Most children either die in the first two years of life or survive in a persistent vegetative state [183]. In addition, *SLC25A22* also plays a role in the epilepsy of infancy with migrating focal seizures (EIMFS), which is a severe developmental and epileptic encephalopathy that also begins in early infancy [188,189].

## 21. Foveal Hypoplasia, 2 (OMIM #609218)

Foveal hypoplasia is characterized by preserved retinal architecture along with the absence of both the retinal foveal pit and the avascular zone. This disorder’s molecular basis is not known. Isolated foveal hypoplasia (in the absence of albinism, aniridia, microphthalmia, or achromatopsia) is exceedingly rare [190]. *SLC38A8* encodes a sodium-coupled neutral amino acid transporter (SNAT8) that is expressed predominantly in the central nervous system and retina with a preference for glutamate as a substrate [190]. Biallelic mutations in *SLC38A8* have been linked to a rare autosomal recessive form of foveal hypoplasia (foveal hypoplasia 2, with or without optic nerve misrouting and/or anterior segment dysgenesis). Clinical manifestations are limited to ophthalmologic symptoms, including low vision and secondary nystagmus [190,191,192,193].

## 22. Cystinosis (OMIM #219800, #219900, and #219750)

Cystinosis is an autosomal recessive lysosomal storage disease caused by intracellular cystine accumulation due to mutations in the *CTNS* gene. This gene encodes cystinosin, which is a cystine/H^+^ symporter that exports cystine out of lysosomes into the cytosol [19,194,195]. A defect in this transporter induces cystine accumulation in many tissues, including the kidney, bone marrow, conjunctiva, thyroid, muscle, brain parenchyma, and others. Cystinosis is classified into three clinical phenotypes: infantile nephropathic cystinosis (#219800), which is the most frequent form, late-onset juvenile nephropathic cystinosis (#219900), and ocular nonnephropathic cystinosis (#219750) [194,196,197,198,199,200].

In infantile nephropathic cystinosis, children are usually asymptomatic at birth and present with the disease between 6–12 months of age. They experience feeding difficulties, vomiting, failure to thrive, and polyuria. Fanconi syndrome is generally present (excessive urinary losses of water, glucose, amino acids, uric acid, phosphate (with hypophosphatemia), sodium, potassium, bicarbonate (leading to hypokalemia, hyponatremia, and metabolic acidosis), and low molecular-weight proteinuria). Hypercalciuria may lead to nephrocalcinosis. Untreated children develop chronic renal failure that progresses to end-stage renal disease in the first decade of life. Involvement of the eye is a primary symptom of cystinosis. Manifestations start with photophobia, which usually appears at two to three years of age [194,196,197]. An ophthalmological examination after one year of life may reveal the presence of corneal cystine crystal deposits [201].

In addition to the infantile form, the juvenile or intermediate form of cystinosis is usually diagnosed during childhood or adolescence. It is characterized by less severe renal symptoms [199]. Nonnephropathic cystinosis involves late-onset photophobia due to corneal cystine crystals but there is an absence of renal disease [200].

Cystinosis should be always suspected in children presenting with Fanconi syndrome since it is the most common inherited cause of Fanconi syndrome. Diagnosis is ascertained by measurement of intra-leucocyte cystine levels and confirmed by molecular analysis of the *CTNS* gene. Most late-onset complications are prevented by oral cysteamine [202]. Regular measurements of intra-leucocyte cystine levels allow for optimization of this treatment. Corneal cystine crystals do not respond to oral cysteamine and require topical administration of cysteamine hydrochloride. Renal transplantation is the treatment of choice for patients with end-stage renal disease [202,203]. Compliance with cysteamine treatment is a major concern in adult patients and could have an impact on the development of neurological and cognitive complications [204]. Autologous transplantation of gene-modified hematopoietic stem cells (HSCs) may be a curative therapy in the future [205].

## 23. Other Possible Inherited Conditions Related to Amino Acid Transporters

A recent study found nine probable pathogenic variants in 11 patients with autism spectrum disorder (ASD) including three in *SLC3A2*, three in *SLC7A5*, and three in *SLC7A8*. *SLC3A2* encodes the heavy subunits and *SLC7A5* and *SLC7A8* of the light subunits of large amino acid transporters (LAT) 1 and 2. LAT1 and LAT2 are responsible for tryptophan and branched-chain amino acids (BCAA) transportation across the blood-brain barrier. They are expressed in both the brain and in blood. Functional studies have been performed, which demonstrate consistently reduced utilization of tryptophan and other large aromatic amino acids. The authors concluded that abnormalities in these transporters are likely to be associated with increased risk of developing ASD [206].

Some cases of dibasic amino aciduria I (OMIM %222690) have been reported. Patients presented with excessive urinary excretion of lysine, ornithine, and arginine. Plasma levels of the amino acid were normal. The inheritance pattern could be autosomal dominant or recessive. Clinical manifestations include hyperactivity, mental retardation, dysarthria, athetosis, and an adverse reaction to phenothiazines. The molecular defect has yet to be identified [207,208,209].

Major depression is a multifactorial disease that has both genetic and environmental risk factors. Recently, the *SLC6A15* gene was proposed as a new candidate gene for vulnerability to stress and major depression. This gene encodes the neutral amino acid transporter B^0^AT2, which is mainly expressed in neurons [210]. In a *SLC6A15* regulatory region, risk allele carriers for a single nucleotide polymorphism (SNP) show altered glutamate levels, hippocampal volume, and hypothalamus-pituitary-adrenal axis activity. These markers are all associated with major depression [210,211].

It is presumed that the transport of aromatic amino acids, such L-tryptophan and other large neutral amino acids, across the ciliary epithelium and into the lens involves a minimum of two transporters, which include LAT2 (encoded by *SLC7A8*) and TAT1 (encoded by *SLC16A10*). Recently, a homozygous mutation in *SLC7A8* has been identified in a family with congenital cataracts [212]. The amino acid transporter TAT1 may have a modifying influence on the development of cataracts [212,213].

## 24. Conclusions

Our work describes different conditions in which a strong association has been found between an amino acid transporter and an inherited metabolic disorder. Many of these inherited disorders have been identified in recent years. Understanding the physiological mechanisms that regulate these transporters, their function, and their distribution will help us better understand the pathophysiology of these diseases. Appropriate clinical and diagnostic characterization of the underlying molecular defect may give patients the opportunity to avail themselves of effective therapeutic options both now and in the future.

## Figures and Tables

**Table 1:** Amino acid transporters included in this review, their function, and involvement in inherited metabolic disease.

SLC	Substrate(s)	Function	Associated Inherited Metabolic Disease
SLC1A1	Glu, Asp, Cys	System XAG	Dicarboxylic aminoaciduria
SLC1A2	Glu, Asp	System XAG	Early infantile epileptic encephalopathy
SLC1A3	Glu, Asp	System XAG	Episodic ataxia type 6
SLC1A4	Glu, neutral AA	System ASC	Spastic tetraplegia, thin corpus callosum, and progressive microcephaly
SLC1A5	Neutral AA	System ASC	
SLC1A6	Glu, Asp	System XAG	
SLC1A7	Glu, Asp	System XAG	
SLC3A1	Cys, dibasic, and neutral AA	Heavy chains of heterodimeric AAT	Cystinuria
SLC3A2	Dibasic and neutral AA	Heavy chains of heterodimeric AAT	
SLC6A5	Gly	Gly transporter	Hyperekplexia 3
SLC6A6	Tau	Tau transporter	Retinal dystrophy
SLC6A7	Pro	Pro transporter	
SLC6A9	Gly	Gly transporter	Glycine encephalopathy with normal serum glycine
SLC6A14	Cationic and neutral AA	System B^0,+^	
SLC6A15	Pro, Met, BCAAs	System B^0^	
SLC6A17	Pro, Gly, Leu, Ala, Glu	System B^0^	Mental retardation, autosomal recessive 48
SLC6A18	Gly, Ala	System Gly	Hyperglycinuria
SLC6A19	Neutral AA	System B^0^	Hartnup disorder, iminoglycinuria, hyperglycinuria
SLC6A20	Pro	System IMINO	Iminoglycinuria, hyperglycinuria
SLC7A1	Lys, Arg, Orn	System y^+^	
SLC7A2	Lys, Arg, Orn	System y^+^	SLC7A2-related argininemia
SLC7A3	Lys, Arg, Orn	System y^+^	
SLC7A4	Cationic AA	System y^+^	
SLC7A5	Leu, Hys, Met, Ile, Val, Phe, Tyr, Trp	System L	
SLC7A6	Lys, Arg, Orn, Hys, Met, Leu	System y^+^L	
SLC7A7	Lys, Arg, Orn, Hys, Met, Leu, Ala, Cys	System y^+^L	Lysinuric protein intolerance
SLC7A8	Neutral AA	System L	
SLC7A9	Cys, dibasic and neutral AA	System b^0,+^	Cystinuria
SLC7A10	Gly, Ala, Ser, Cys, Thr	System ASC	
SLC7A11	Glu, Asp, Cys	System y^+^	
SLC7A12	Gly, Ala, Ser, Cys, Thr	System ASC	
SLC7A13	Glu, Asp, Cys	Glu/Asp/Cys transporter	Cystinuria
SLC7A14	Arg, Lys, Orn	System C	Retinitis pigmentosa 68
SLC16A10	Trp, Tyr, Phe	System T	
SLC17A6	Glu	Vesicular Glu transporter	
SLC17A7	Glu	Vesicular Glu transporter	
SLC17A8	Glu	Vesicular Glu transporter	Deafness, autosomal dominant 25
SLC25A2	Lys, Arg, Hys, Orn, Cit, ADMA	Orn/Cit carrier	
SLC25A12	Asp, Glu	Asp/Glu carrier	Early infantile epileptic encephalopathy, 39
SLC25A13	Asp, Glu	Asp/Glu carrier	AGC2 deficiency
SLC25A15	Lys, Arg, Hys, Orn, Cit	Orn/Cit carrier	Hyperornithinemia-hyperammonemia- homocitrullinuria (HHH) syndrome
SLC25A18	Glu	Glu carrier	
SLC25A22	Glu	Glu carrier	Early infantile epileptic encephalopathy, 3
SLC25A29	Arg, Lys, Orn, Hys	Basic AA transporter	
SLC32A1	Gly, GABA	Vesicular Gly/GABA transporter	
SLC36A1	Gly, Pro, Ala	Proton AA symporter	
SLC36A2	Gly, Pro, Ala	Proton AA symporter	Iminoglycinuria, hyperglycinuria
SLC36A3	Gly, Pro, Ala?	Proton AA symporter	
SLC36A4	Pro, Trp, Ala	Proton AA symporter	
SLC38A1	Gly, Alan, Ser, Cys, Gln, Asn, Hys, Met, Thr, Pro, Tyr, Val	System A	
SLC38A2	Gly, Pro, Ala, Ser, Cys, Gln, Asn, Hys, Met	System A	
SLC38A3	Gly, Pro, Ala, Ser, Cys, Gln, Met, Hys, Lys, Arg	System N	
SLC38A4	Gly, Ala, Ser, Cys, Gln, Asn, Met	System A	
SLC38A5	Gln, Asn, Hys, Ala	System N	
SLC38A7	Gln, Ala, Hys, Asn, Ser	System N	
SLC38A8	Gln, Ala, Arg, Hys, Asp	System A	Foveal hypoplasia, 2
SLC38A9	Gln	Lysosomal Gln transceptor	
SLC38A10	Gln, Ala, Glu, Asp, Ser	System A	
SLC43A1	Leu, Ile, Met, Phe, Val	System L	
SLC43A2	Leu, Ile, Met, Phe, Val	System L	
SLC66A4	Cys and cystathionine	Lysosomal Cys transporter	Cystinosis

**Table 2:** Amino acid transporters with associated inherited metabolic disease: name of the amino acid transporter, gene/locus, location, phenotype MIM number, inheritance, and main clinical manifestations.

Amino Acid Transporter	Gene/Locus	Location	Associated Inherited Metabolic Disease	Phenotype MIM Number	Inheritance	Clinical Manifestations
EAAT3	*SLC1A1*	9p24.2	Dicarboxylic aminoaciduria	222730	AR	Possibly benign in most cases. Associated with OCD and schizophrenia
EAAT2	*SLC1A2*	11p13	Early infantile epileptic encephalopathy	617105	AR/AD	Severe early-onset epileptic encephalopathy
EAAT1	*SLC1A3*	5p13.2	Episodic ataxia type 6	612656	AD	Episodic ataxia, seizures, migraine, alternating hemiplegia
ASCT1	*SLC1A4*	2p14	Spastic tetraplegia, thin corpus callosum, and progressive microcephaly	616657	AR	Spastic tetraplegia, thin corpus callosum, and progressive microcephaly
rBAT	*SLC3A1*	2p21	Cystinuria	220100	AR/AD	Cystine stones
GLYT2	*SLC6A5*	11p15.1	Hyperekplexia 3	614618	AR/AD	Startle reflex, generalized muscle stiffness, sudden infant death
TAUT	*SLC6A6*	3p25.1	Retinal dystrophy	-	AR	Early-onset atypical panretinal degeneration
GLYT1	*SLC6A9*	1p34.1	Glycine encephalopathy with normal serum glycine	617301	AR	Early-onset encephalopathy with severe hypotonia, dysmorphic features, and abnormal antenatal findings
NTT4	*SLC6A17*	1p13.3	Mental retardation, autosomal recessive 48	616269	AR	Intellectual disability with progressive tremor, speech impairment, facial dysmorphism, and behavioral problems
B^0^AT1	*SLC6A19*	5p.15.33	Hartnup disorder Hyperglycinuria Iminoglycinuria	234500 138500 242600	AR AD AR/AD	Pellagra-like dermatitis, intermittent cerebellar ataxia, neuropsychiatric symptoms Possibly benign Possibly benign
SIT1	*SLC6A20*	3p21.31	Iminoglycinuria Hyperglycinuria	242600 138500	AR/AD AD	Possibly benign Possibly benign
CAT-2	*SLC7A2*	8p22	Argininemia SLC7A2-related	-	AR	Unknown
y^+^LAT1	*SLC7A7*	14q11.2	Lysinuric protein intolerance	222700	AR	Hyperammonemia, protein intolerance, growth failure, renal disease, lung disease, immunological alterations
b^0,+^ AT	*SLC7A9*	19q13.11	Cystinuria	220100	AR/AD	Cystine stones
SLC7A14	*SLC7A14*	3q26.2	Retinitis pigmentosa 68	615725	AR	Retinitis pigmentosa/Leber congenital amaurosis
VGLUT3	*SLC17A8*	12q23.1	Deafness, autosomal dominant 25	605583	AD	Slowly progressive high frequency sensorineural hearing loss
AGC1	*SLC25A12*	2q31.1	Early infantile epileptic encephalopathy, 39	612949	AR	Progressive encephalopathy, hypotonia, microcephaly, myoclonic epilepsy
AGC2	*SLC25A13*	7q21.3	Neonatal intrahepatic cholestasis Citrullinemia type II	605814 603471	AR	Neonatal intrahepatic cholestasis with persistent jaundice Neuropsychiatric symptoms, ammonia intoxication
ORC1	*SLC25A15*	13q14.11	Hyperornithinemia-hyperammonemia- homocitrullinuria syndrome	238970	AR	Episodic hyperammonemia and neurological symptoms
GC1	*SLC25A22*	11p15.5	Early infantile epileptic encephalopathy, 3	609304	AR	Myoclonic epilepsy, progressive microcephaly, hypotonia
PAT2	*SLC36A2*	5q33.1	IminoglycinuriaHyperglycinuria	242600 138500	AR/ADAD	Possibly benignPossibly benign
SNAT8	*SLC38A8*	16q23.3	Foveal hypoplasia, 2	609218	AR	Low vision, secondary nystagmus
CTNS	*SLC66A4 (CTNS)*	17p13.2	Cystinosis	219800 219900 219750	AR	Fanconi syndrome, photophobia, neurological deterioration
